# Beliefs in the Future as a Positive Youth Development Construct: A Conceptual Review

**DOI:** 10.1100/2012/527038

**Published:** 2012-04-24

**Authors:** Rachel C. F. Sun, Daniel T. L. Shek

**Affiliations:** ^1^Faculty of Education, The University of Hong Kong, Hong Kong; ^2^Department of Applied Social Sciences, The Hong Kong Polytechnic University, Hong Kong; ^3^Public Policy Research Institute, The Hong Kong Polytechnic University, Hong Kong; ^4^Department of Sociology, East China Normal University, Shanghai, China; ^5^Kiang Wu Nursing College of Macau, Macau; ^6^Division of Adolescent Medicine, Department of Pediatrics, Kentucky Children's Hospital, University of Kentucky College of Medicine, Lexington, KY 40506, USA

## Abstract

Beliefs in the future are an internalization of hope and optimism about future outcomes. This paper reviews and compares several theories of hope and optimism and highlights the features constituting beliefs in the future. This paper points out that beliefs in the future include a series of goal-directed thoughts and motivation, such as setting up valued and attainable goals, planning pathways, and maintaining self-confidence and mastery, so as to keep adolescents engaged in the pursuit of goals. This kind of personal mastery, together with sociocultural values, family, school, and peers are the antecedents leading to beliefs in the future, which is related to adolescents' well-being and positive development. In order to cultivate adolescents' beliefs in the future, enabling their ability to manipulate goal-directed thoughts and motivation and providing a supportive environment including their family, school, peers, and the society are recommended.

## 1. Background

Catalano et al. [1] defined “beliefs in the future” as “an internalization of hope and optimism about possible outcomes.” That is, beliefs in the future entail the concepts of hope and optimism and the ability to internalize both in anticipating future outcomes. They play a vital role in the growth of adolescents who are encountering an increasing number of future life options, such as studies, careers, and heterosexual relationships [2], that need them to set up personal goals. Research findings showed that adolescents aged between 12 and 19 were able to generate various personal goals relating to school, future trajectory, material, free time, relationship, self, health, and body, though there were differences in the goal content and pursuit between younger and older adolescents [3]. It is because the progressive cognitive development enables adolescents to conduct reasoning, test hypotheses, set personal goals, and plan more realistically [4, 5], which allows them to play an active role in envisaging and manipulating their future possibilities. Moreover, the cognitive advancement allows adolescents to evaluate goal attainment, generate alternative pathways, and modify goal planning, that are closely interlinked with their self-efficacy, optimism, resiliency against adversities, and persistence [6]. As such, beliefs in the future, as well as hope and optimism, are regarded as important personal strengths in positive psychology [7, 8] and positive youth development [1].

In regard of this, this paper reviews and compares several theories of hope and optimism, and highlights the features constituting beliefs in the future. It looks at the antecedents leading to beliefs in the future and the relationships of hope and optimism to adolescents' well-being and positive development. As hope and optimism share a common theme of future orientation that keeps one engaged in the pursuit of goals [9, 10], this paper translates hope and optimism into a series of goal-directed thoughts and motivation so as to enable adolescents to internalize both in expecting future outcomes. It also discusses several ways to nurture adolescents' beliefs in the future.

## 2. Definition of Beliefs in the Future

Based on the definitions given by Catalano et al. [1] and Sun and Lau [11], hope and optimism constitute “beliefs in the future” that include (i) goal-directed thoughts, such as setting up valued and attainable goals and planning primary and alternative goal-directed pathways and (ii) goal-directed motivation, such as self-confidence and mastery that are derived from positive appraisal of one's capability and effort. These thoughts and motivation influence each other reciprocally in the process of goal pursuit and would rejuvenate when the goals are successfully attained.

## 3. Hope

There are two lines of research in understanding the definition of hope. One is the emotion-based model which states that hope is “an emotion that occurs when an individual focuses on an important future outcome that allows little personal control, so the person is unable to take much action to realize the outcome” [12, page 348]. In this perspective, hope is conceptualized as an emotion, usually a positive affect that keeps adolescents engaged with the future outcomes, though one may not control the outcome. As such, the future outcome needs to be valuable, so that one can carry positive expectancy despite the likelihood of occurrence being low [13].

Unlike the emotion-based model of hope, the cognitive-motivation-based model argues that adolescents can control future outcomes as hope is “the perceived capability to derive pathways to desired goals, and motivate oneself via agency thinking to use those pathways” [14, page 249]. Hope is perceived as a trait comprising the will and the ways to attain the goals [15]. Such a goal-directed hope is acknowledged because one can exercise a certain level of mastery while anticipating future possibilities—“yet hope is not necessarily a bad thing because mental time travel to the future can sometimes affect the present… It can set goals, develop plans, impel action, and thus transform the hypothetical future into reality” [16, page 140]. According to Snyder [14], goals, pathways (planning to meet goals), and agency (goal-directed energy) are the three important cognitive and motivational components in the trilogy concept of hope. These three components are interactional, and adolescents can exercise their personal control in setting goals and manipulating their pathway and agency thinking in the goal-pursuit process. The role of emotion is not completely denied. In fact, successful goal attainment, as well as positive affect that resulted from successful goal attainment, feedback, and energize a series of goal-directed thoughts, motivation, and behaviors and further contribute to forward flow of hope [6].

### 3.1. Goal

There are different types of goals serving different levels of purposes [14]. Some goals are positive and approachable, such as those (a) going to be reached for the first time, (b) sustaining of a present goal, and (c) furthering upon which already has been initiated. On the other hand, some goals are negative including those (a) stopping something before it happens and (b) delaying the unwanted. These goals are purposes to be achieved, which generate a series of actions to attain the goals; if not, they are simply desires. Adolescents need to identify the purpose and value of what they are doing and to set up realistic goals of which they can achieve and succeed [17]. The fact is having valued and attainable goals can keep adolescents engaged and being confident in accomplishing these goals [18]. Otherwise, adolescents are less motivated in goal pursuit particularly when there is either too low or too high probability of success. In short, it is essential to set up goals that are valued enough to arouse motivation to attain (i.e., agency thinking), as well as realistic enough to elicit concrete planning (i.e., pathway thinking).

### 3.2. Pathway Thinking

Pathway thinking is “goal-directed thoughts” referring to adolescents' cognitive ability to plan feasible ways to meet their desired goals, as well as to generate alternative pathways when they encounter obstacles in goal attainment [6, 19]. It is evolving throughout one's trials and errors, as Snyder noted, “pathway thinking should become increasingly refined and precise as the goal pursuit sequence progresses toward the goal attainment” [14, page 251]. Trait was found to make a difference in which high-hope people are more quickly to set up and refine their primary routes to reach their goals in an effective manner than low-hope people [5]. Nevertheless, pathway thinking can be made explicit and translated into a series of goal-setting and goal-planning skills that enable low-hope people to make improvement and high-hope people to make advancement.

With reference to the advancement in abstract thinking and reasoning [4, 5], adolescents are capable of setting up concrete and realistic goals for planning feasible ways of goal attainment. For instance, adolescents can set up achievable short-term goals in which a series of connected short-term goals would become a long-term goal or set up a long-term goal which can be broken down into several consecutive short-term goals to make goal attainment more realistic. Moreover, they can focus on a single goal or set up multiple goals that allow flexibility and reservation when the pursuit of a goal failed. Most importantly, they have to be informative of the situation, be evaluative of their ability within the time and resource constraints/availability, and be able to prioritize multiple goals in accordance with their values. For such global thinking and reasoning help setting up measurable and manageable goals, that not only enable adolescents to plan feasible pathways and alternatives to attain within one's capability, but also allow them to evaluate successes or failures which become reference points for future modification and progress of their pathway thinking in the process of goal attainment.

### 3.3. Agency Thinking

Agency thinking is “goal-directed motivation” referring to adolescents' appraisal of their capability to move along the pathways to achieve their goals, which is associated with one's confidence, mental will power, and perseverance in the course of goal attainment [6, 19]. Agency thinking is similar to self-efficacy that refers to “people's beliefs about their capabilities to produce designated levels of performance that exercise influence over events that affect their lives” [20, page71]. According to Bandura [20], adolescents who had higher appraisal of their capabilities tend to set higher goals and are more committed to plan courses of action to realize those goals. Hence, the central inquiry is how adolescents appraise their capabilities, for such an appraisal would affect their goal-pursuit motivation and behavior. Generally speaking, one way of appraising one's capability to reach a goal is to derive from past experiences—attribution of the causes of successes and failures [21]. If adolescents attribute their past successes to their “ability” (an internal and stable factor), it definitely enhances their self-efficacy and confidence in achieving future goals. However, if they attribute their past failures to their ability which is also an uncontrollable factor, they will become frustrated and hopeless when they think it is hard to change the “cause” (i.e., ability) and so as the negative “outcome” (i.e., failure). On the other hand, if adolescents attribute their past successes and failures to causes within internal locus of control such as “effort” [21, 22], the sense of mastery would energize them to be more persistent to achieve success or to avoid failure. Moreover, the beliefs that one can succeed (because of one's ability and/or effort) can be self-fulfilling and further motivate adolescents to exercise control in the goal-pursuit process. Therefore, it is important to have an optimal appraisal of one's ability and effort in achieving goals, for such agency thinking is motivational that keeps adolescents cognitively and behaviorally engaged in creating pathways, as well as generating alternatives in times of encountering obstacles.

 In sum, according to the cognitive-motivation-based model [14], hope is future-oriented thoughts and motivation, embracing (i) setting up valued and attainable goals, (ii) planning primary and alternative goal-directed pathways, and (iii) having positive appraisal of one's capabilities and effort, that benefit goal pursuit.

## 4. Optimism

Optimism refers to positive expectancy about the future [23]. It is a kind of personality that has an obvious benefit in psychological and physical well-being [24]. However, unrealistic optimism can be detrimental if people only expect good things to happen and rarely prepare themselves to cope with the situations, or do not make an accurate evaluation of their life and overanticipate to having a brighter future [25]. It reveals that optimism embraces some cognitive components, such as a goal or an expectation, that regulate behavior. Optimism can then be learnt [26] while it is dispositional, for enabling goal attainment, positive growth and well-being, and vice versa.

Based on the conceptual framework of expectancy-valued models of motivation, Carver and Scheier [18] pointed out that optimism embraces (i) valued and attainable goal which keeps adolescents engaged in the process of goal attainment and (ii) a sense of confidence which encourages adolescents to carry out effortful behaviors, even in the face of adversity. Research findings showed that optimistic expectations for future goal pursuit influenced immediate acts [27] and helped in overcoming anticipated obstacles in goal pursuit [28]. Hence, this positive expectancy about the future is similar to goal-directed motivation (agency thinking) [14] and self-efficacy [20] discussed in the previous section. However, the difference is positive expectancy alone elicits goal-directed behaviors in the conception of optimism, whereas both pathway thinking and agency thinking are equally important in bringing forth goal-directed behaviors in the conception of hope. Another difference is optimists engage their efforts to reach goals as long as there is positive expectancy for eventual success, whereas self-efficacious people drive towards their goals as long as they believe they can.

Alternatively, Seligman et al. [29] adopted an attribution explanatory model to elucidate optimism. Optimistic adolescents explain good events as having permanent and pervasive causes, hence they are confident and will try harder to achieve positive outcomes in the future. In addition, optimistic adolescents make realistic judgment on one's responsibility when things go wrong. Even when they blame themselves for the faults, they will attribute them to temporary, specific, and internal causes, such as effort that they can adjust. Again, like the agency thinking of hope, this positive and realistic explanatory style generates self-confidence and a sense of mastery to achieve, to make changes, and to overcome challenges in the process of goal attainment.

 On the other hand, pessimism, which is at the other end of the pole, is not totally detrimental to goal attainment. Hazlett et al. [30] found that people who simply look for maintenance, safety, and security performed better when they preferred pessimistic forecasts, whereas people who are motivated for attainment, growth, and advancement performed better in goal pursuit when they preferred optimistic forecasts of their future. That means, different types of goals can be achieved, provided that one's motivation orientation (preventive or promotional) and self-regulatory preferences (pessimistic or optimistic) are matched. In positive youth development, thus, it is crucial to encourage adolescents to pursuit growth and advancement and to adopt a positive self-regulation. As such, optimism refers to positive expectancy about the future [23], including setting valued and attainable goals and developing a sense of confidence that can be generated from positive and realistic attribution of one's experiences.

## 5. Relationship between Hope and Optimism

In contemporary research studies, the psychometrically validated Hope Scale [31] and Life Orientation Test [32] are commonly used to measure hope and optimism, respectively, among children and adolescents. These two scales were found to have convergent validity, and were highly recommended for research use after comparing with other instruments [33]. In addition, there were several empirical findings showing that both hope and optimism are related yet distinct constructs [9, 34], predicting life satisfaction [35] and well-being [36]. All these lend support to the conceptual understanding that both hope and optimism are closely interrelated concepts of future orientation and positive expectancy [10], and thus hope and optimism are regarded as the components of beliefs in the future, that contributes to adolescent development and well-being.

## 6. Beliefs in the Future and Adolescent Developmental Outcomes

Relating beliefs in the future as goal-directed thoughts and motivation, a review showed that adolescents' goal content and pursuit are connected to their behavior, health, and well-being [37]. For the goal content, research findings showed that goals related to learning and mastery [38] and intrinsic values of self-acceptance and affiliation [39] had stronger contribution to student well-being. In the goal-pursuit process, students having lower goal-related self-efficacy, greater goal-attainment difficulty, and frustration were found to have poorer well-being [3]. In contrast, resilience to challenges, such as using emotional regulation, goal regulation and social support, was contributory to student well-being when encountering adversity in the goal-pursuit process [40].

Moreover, hope is considered as one of the character strengths contributory to life satisfaction among youth [41], predicting self-esteem [42] and positive affect [7], lowering internalizing behavior, and moderating the negative effects of life stress on adolescent psychological well-being [7]. Furthermore, hope was found to be a strong predictor of academic achievement over years among high school students in Australia [43] and college students in the United States [44]. In particular, pathway thinking was found to be a unique predictor of academic achievement, when controlling the effects of intelligence, personality, and previous academic achievement among a group of university undergraduate students in the United Kingdom [45]. On the other hand, agency thinking was found to be a strong and consistent predictor of life satisfaction across college students and adults in the United States [46].

Similarly, optimism is regarded as a personal strength [8], predicting life satisfaction among Taiwan adolescents [47] and mediating the relationship between meaning in life and psychosocial problems among Hong Kong adolescents [48]. Both optimism and hope were found to significantly predict life satisfaction and negatively predict depression among Singaporean adolescents [35], whereas optimism and life satisfaction were found to negatively predict depression among Hungarian adolescents [49]. Furthermore, a combination of both optimism and hope, as a goal attitude, was found to be a significant predictor of academic grade among university students in the United States [50].

Research studies comparing students with different levels of hope showed that students having high hope felt more inspired, energized, confident, and challenged by their goals [31], had higher levels of self-worth and lower levels of depression [51, 52], and had higher academic achievement [53] when compared with students having low hope. In particular, middle and high school students having high hope were found to have less school maladjustment and emotional distress, higher personal adjustment, life satisfaction, and academic achievement, and more participation in school extracurricular activities [54], and be less likely to drop out even though they were at risk for dropping out [55]. Also, college students with high hope were found to have greater problem-solving abilities and coping than low-hope students [56]. In the same vein, when compared with pessimistic students, studies found that optimistic students had lower stress levels and fewer depressive and physical symptoms [57] and were more able to use a variety of problem-solving and emotion-focused strategies to cope with stress [58]. It was also found that optimists had more positive evaluation of their past and present and made more realistic anticipation of the future than pessimists [25]. Like hope, optimism has positive associations with adolescents' psychological and physical health and adaptive coping [24].

## 7. Antecedents of Beliefs in the Future in Adolescents

A survey of the literature showed that there are various sociodemographic antecedents (e.g., age, gender, ethnicity, socioeconomic status, family, peers, school, and social and political environment) and individual's psychological and behavioral problems affecting adolescents' goal content and pursuit [37]. According to the human ecological model [59, 60], adolescents' beliefs in the future are products of the environmental influences and their own manipulation of such influences. As the role of “personal mastery” [14, 23, 30] was discussed in the earlier parts of the paper, the following environmental factors (i.e., sociocultural values, ideologies, family, school, and peers influences) affecting adolescents' beliefs in the future are discussed.

### 7.1. Sociocultural Values and Ideologies

Some researchers highlighted that there are differences in the conceptualization of optimism between the Western and Chinese cultures (e.g., [61]). Therefore, when adopting the Western conceptions of hope and optimism to understand Chinese adolescents' beliefs in the future, the influences of Chinese cultural values and social contexts cannot be overlooked. In Chinese culture, high value is placed on academic success as it is associated with prospective studies, rewarding careers and wealth [62]. These messages are transmitted both explicitly and implicitly through mass media, comparison and competition, social communication, and expectation. When adolescents internalize these values, they tend to perceive getting academic success and prosperous careers as their learning goals and thus are intrinsically motivated to study well so as to achieve. They put effort to attain their goals, because such a personal control is highly acknowledged in the Chinese proverbs, like “jin ren shi an tian ming” (try our best but leave the fate to heaven) and “cheng shi zai tian, mou shi zai ren” (men can plan but the outcomes are determined by heaven), albeit the ultimate success is determined by fate. Several beliefs, for example, “failure is the mother of success” and “there is light at the end of the tunnel.” also keep adolescents being optimistic and autonomous in the process of goal attainment, even when encountering difficulties or failures. Therefore, with unique values and ideologies, several studies found that adolescents living in different sociocultural contexts had different goal orientations [63, 64].

Moreover, sociocultural expectation of gender roles, which is traditionally rooted as well as being reinforced by mass media, also affects adolescents' goal orientation, aspirations, and perceived pathways. Research studies showed that there were significant gender differences in adolescents' goals related to future careers, education, family, marriage, leisure activities, and properties [65–67], and girls tended to report having more obstructed goals, greater goal frustration and lower goal-related self-efficacy than boys [3]. These might be related to the gender stereotypes in careers, such as “men are breadwinners; women are housewives.” However, Greene and DeBacker [68] indicated that there has been a change in women's roles, and so do their future orientations that are not restricted to family aspect only, but include both family and career expectations simultaneously.

### 7.2. Family Influences

In family, parental support, involvement, nurturance, attainment beliefs, and aspirations for the children were revealed to have significant influences on adolescents' goal setting and attainment [37]. Fitzsimons and Finkel [69] also added that interpersonal processes played a crucial role in affecting adolescents' goal setting and pursuit. Taking parenting as an illustration, if parents are demanding but responsive to the growing needs of their children, it will facilitate their children to set achievable goals and find plausible ways to attain. It was evidenced by research findings that perceived parental authoritativeness (demanding but responsive) was related to higher levels of hope over years, whereas perceived parental authoritarianism and permissiveness did not show significant correlations with hope [70]. Another longitudinal research study also found that optimism mediated the predictive effects of authoritative parenting on students' self-esteem, depression, and school adjustment [71]. In short, parental acceptance and support are essential in fostering adolescents' beliefs in their future.

On the other hand, negligence, conflicts, and uncontrollable traumatic events in family (such as being abused) will dampen adolescents' hope and optimism. For instance, research studies showed that Chinese adolescents who had more parent-child conflicts were likely to have feelings of hopelessness [72], whereas Arab adolescents who were physically and psychologically maltreated in family or witnessed violence and aggression between parents reported having hopelessness, low self-esteem, and psychological adjustment problems [73, 74].

### 7.3. School Influences

Between schools, differences in the environment and educational tracks were found to have different influences on adolescents' goal setting and attainment [37]. Within a school, differences in educational and developmental preparation in different grade levels were related to the age differences in goal setting, in which younger students tended to focus more on school goals whereas older students tended to focus more on future trajectory goals and have higher levels of goal-related self-efficacy [3]. In addition, social comparison, which is inevitable under the competitive learning environment and assessment system, can affect students' perceptions of their future. In particular, academic failure would lead to learned hopelessness, particularly among academically low achievers who had already studied hard [75].

Nevertheless, school-based intervention programs, for instance, the Penn Optimism Program [76] and the Penn Resiliency Program [77], were found to be effective in increasing students' levels of hope, optimism, and resiliency and reducing their levels of depression and anxiety [78]. Other hope-based interventions designed to enhance the goal-directed thinking in children [79], and at the same time in collaboration with parents and teachers [80], were also found to be effective in enhancing children's hope, life satisfaction, and self-worth. Miller et al. [81] thus proposed that promotion of hope and optimism in school is a promising way of wellness enhancement for reducing disorders and fostering health among students.

### 7.4. Peer Influences

Peer support was shown to contribute to adolescents' goal pursuit [82] and hope [83]. However, students who did not have adequate peer and family support and positive perceptions about school and oneself were more likely to have hopeless feelings [84]. Furthermore, being victimized by peers in school would also lead to social hopelessness and thus suicidal ideation, though family support could act as a buffer [85]. It indicated that having adequate social support, ranging from one to multiple networks including family, peers, and school, is pertinent for maintaining adolescents' psychological well-being and positive development [1].

## 8. Cultivating Adolescents' Beliefs in the Future

There are several ways to foster students' beliefs in the future. First, schools can arrange curricula-based programs [76, 77, 86, 87], since these programs were evidenced to enhance students' hope, optimism, and well-being. These programs can focus on strengthening adolescents' competence and resilience, such as setting up valued and attainable goals, planning primary and alternative pathways, and appraising one's capability and effort positively. Moreover, these programs can incorporate some positive cultural values and ideologies to cultivate adolescents' optimistic and hopeful orientation towards the future. At the same time, schools need to develop a goal-directed learning environment and arrange more opportunities both inside and outside the classrooms for students to master the learnt skills and maintain their aspirations. Besides, developmental group work can be carried out to promote students' beliefs in the future, when more intensive intervention is needed. Of course, materials in the curricula-based programs can be used in the group work context. In addition, specialized intervention programs such as adventure-based counseling can be attempted. All these programs require the joint hands between teachers and social workers and the utilization of peers as a supportive resource.

Second, career education and guidance [88] can be provided primarily by teachers and social workers, with the support of the potential mentors in the community. It not only guides students to link up school learning with one's future career in the society, but also widens their career horizon. It is believed that early career awareness and preparation can stimulate students' future orientation. Also, it can let students to have better self-understanding and self-acceptance, which help tackling gender stereotypes and myths about career choices and pathways.

Third, school teachers and social workers can collaborate with students' parents so as to encourage parental involvement and support in fostering adolescents' beliefs in the future. Parenting skills workshops can be attempted to remind parents to accept and respect their children of who they are and what they could achieve, be responsive to their children's growing needs and be demanding but avoid setting unrealistic expectations. All in all, adolescents themselves, as well as their family, peers, school, and the community, can jointly work together to nurture adolescents' beliefs in the future.

## 9. Conclusion

Promotion of beliefs in the future for positive youth development deserves greater attention since there is growing research evidence demonstrating its positive effects on adolescent well-being. Noting that hope and optimism are the two core components of beliefs in the future, it is necessary to help adolescents to internalize both hope and optimism by facilitating them to manipulate their goal-directed thoughts and motivation and by providing a supportive environment including their family, school, peers and the community.
